# Youth-led mental health promotion in Pakistan informed by intergenerational resilience

**DOI:** 10.1177/17579759251342821

**Published:** 2025-07-01

**Authors:** Panos Vostanis, Sajida Hassan, Syeda Zeenat Fatima, Michelle O’Reilly

**Affiliations:** 1 Criminology, Social Policy and Sociology, University of Leicester, UK; 2 Icon for Child and Adult Nurturing (ICAN), Karachi, Pakistan

**Keywords:** adolescents and youth, mental health promotion, intergenerational, Majority World countries

## Abstract

Interventions informed by intergenerational resilience have shown positive effects on youth mental health. In Majority World countries, however, mental health promotion remains constrained by stigmatizing beliefs and limited resources. This study explored multiple stakeholder perspectives of how intergenerational learning was incorporated in youth-led mental health promotion in Pakistan. Fourteen youth peer educators co-designed a mental health promotion programme with family advisers and facilitated 11 workshops in two disadvantaged areas of Karachi. Of the 304 workshops participants, a sub-sample of 63 youth, mothers, teachers and peer educators attended 11 focus groups. Established themes related to the process that enabled intergenerational learning, notably participatory activities, and how such learning could be transferred to resilience-enabling systems by mobilizing communities. A multidimensional resilience framework that draws upon intergenerational experiences can usefully inform youth mental health promotion, especially in resource-constrained settings. Co-production with holders of local knowledge can engage and empower communities.

## Introduction

Trauma exposure can be transmitted to future generations, referred to as intergenerational trauma, through events like loss, abuse and gender-based violence (1), mediated by disrupted attachment relationships and structural inequalities (2). These factors may in turn adversely influence mental health (3). In collective societies (Majority World Countries (MWCs)), additional cultural and historical influences on shared trauma following political or war conflict (4) interact with exposure to poverty, violence or impaired parenting capacity (5). Nevertheless, the trauma cycle can be broken by enhancing intergenerational resilience through social adaptation, protection of cultural identity and positive parenting (6,7). Consequently, resilience-building factors were incorporated in intergenerational learning interventions. These were informed by different age groups sharing experiences of both adversities and coping with those in different historical periods, thus identifying commonalities in building resilience across such contexts. Delivery approaches included physical, reading, arts, life skills, interpersonal and digital activities (8). Despite the heterogeneity of these approaches, promising findings emerged on promoting mental health across age groups (9). These were found to be particularly important for children and youth, who heard stories from grandparents or other older adults on how they had dealt with severe and prolonged stressors such as poverty, natural disasters, war conflict or discrimination, before transferring lessons to their current life challenges. Community members were shown to be ideally positioned in promoting mental health informed by intergenerational resilience, as carriers of local knowledge and wisdom on how to understand, process and reframe trauma across different generational contexts (10).

Community-based mental health promotion programmes are important in challenging stigma, enhancing knowledge and encouraging help-seeking. In MWC resource-constrained settings, with continuities of conflict and disadvantage, the incorporation of intergenerational learning is important in breaking the cycle of poverty and mental ill health, especially when led by peer educators who can engage communities (11). Whilst all community volunteers or peer educators are holders of lived experience and unique local wisdom, youth are particularly well positioned to process different intergenerational perspectives *and* engage other youth. Overall, previous research explored how intergenerational learning, and resilience, can inform mental health promotion and interventions. A separate body of research demonstrated the potential benefits of community volunteers such as youth peer educators providing mental health promotion. The rationale of this study was to *integrate* the two research fields by establishing multiple stakeholder perspectives of how intergenerational learning was incorporated in youth-led mental health promotion in Pakistan.

## Methods

The aim of the study was addressed by the following research questions:

1. In what ways can intergenerational experiences of resilience inform youth-led mental health promotion in disadvantaged communities of Pakistan?
2. What types of intergenerational delivery approaches can engage participants?
3. What are the benefits and challenges of a youth-led intergenerational learning approach for mental health promotion?

The project consisted of two phases: phase 1 involved the design of a mental health promotion programme, was reported in (12) and is summarized below; whilst phase 2 involved the implementation process and is reported in this paper.

### Family-informed design of mental health promotion

The study was conducted in two disadvantaged areas of Karachi: Lyari in South (661,926 population) and Lines (730,000 population) in East Karachi (13). It was hosted by a non-governmental social enterprise providing child psychosocial support, which recruited 11 family units as intergenerational advisers. Each family unit consisted of one young person (female 14–18 years), one mother (38–45 years) and one grandmother (55–70 years). Fourteen peer educators were community volunteers from the social enterprise network, all female, and aged 17–28 years. Their backgrounds were: Educational Psychologist, Teacher, Artist, Clinical Psychologists (two), University Graduates (Psychology and Sociology), university students (Psychology – three, Computer Sciences, pre-Medical) and high school graduates (two). Peer educators attended 12 mental health training sessions and facilitated three participatory consultation workshops with the family advisers on how intergenerational experiences of resilience could be incorporated in mental health promotion.

### Implementation of mental health promotion

The objectives were to promote emotional literacy, recognition of strengths, and development of adaptive coping strategies, especially by drawing on intergenerational experiences of resilience. Youth peer educators were considered as better positioned than adults to engage different generational groups. The objective was to reach as many age groups as possible. Following the consultation phase (12) and at this preliminary stage, however, it was considered preferable to deliver mental health promotion to separate rather than mixed groups – for example, because of tensions between adults and youth, or between grandmothers and daughters-in-law, which could not be safely addressed within this time framework. In each area, workshops involved one group of youth (female or male, because of single-gender schools), mothers, grandmothers and teachers. For logistical purposes, grandmothers could be involved in only one area and could not attend focus groups. An additional group of religious teachers was included in one area, following teachers’ request. In total, 11 workshops were facilitated. Participants’ numbers and age range are presented in Table 1. Although the sampling framework was not restricted in terms of gender, participants were largely female, except for two male youth groups. The reason was mainly cultural in not involving mixed gender groups and partly low male adult engagement, which is common in child mental health research.

**Table 1.** Profile of mental health promotion participants.

Area	Target group	Number (*N* = 304)	Age range	Focus group sub-sample (*n* = 63)
1	Female youth	45	14–17	5
1	Male youth	20	14–17	7
1	Mothers	25	25–40	5
1	Grandmothers	17	45–75	-
1	Teachers (female)	50	28–40	7
1	Religious teachers (female)	30	25–48	5
2	Youth (female)	25	14–17	5
2	Youth (male)	25	14–17	5
2	Mothers (single)	30	25–40	5
2	Teachers (female)	30	21–36	5
1	Peer educators (female)	7	17–27	7
2	Peer educators (female)	7	19–28	7

Peer educators delivered four-hour workshops in community centres for parents and grandparents, and in schools for youth and teachers. The objective was to develop an understanding of positive mental health and wellbeing (in contrast with sole focus on mental health problems and illness) and to promote strategies of mental health self-care and support for children and youth. Each workshop addressed the following topics: conceptualization of mental health and wellbeing, attitudes and beliefs towards mental health, perceived risk factors for mental health problems, protective factors when faced with stressors, and development of adaptive coping strategies. Risk and protective factors were considered across socioecological systems (individual, family, community, society). Delivery was participatory, with experiential and problem-solving activities. The difference from generic mental health promotion events was the use of examples, lessons and experiences from participants and facilitators on how different generations could learn from each other in building resilience in response to individual and collective stressors. The research team and peer educators developed a common template for all workshops; however, facilitators were encouraged to adapt activities to their target group. Examples of intergenerational activities are presented in Appendix 1.

### Data collection and analysis

Focus groups engage stakeholders in ‘collective conversations’ to share experiences, insights and perspectives (14). Through purposive sampling, we invited a sub-sample of participants from each workshop and all peer educators to attend focus groups. Participant numbers (5–7 per focus group) are presented in Table 1. In total, 11 focus groups were facilitated by an independent researcher of Psychology background and were held at the same community centres or schools as the workshops. Each focus group lasted 60–90 min. The topic guide explored participants’ experience of workshops, particularly in relation to intergenerational aspects, whether they had applied new knowledge, and recommendations on how these could be scaled up in future. Discussions were in Urdu and were audio-recorded. Ethics approval was granted by the University of Leicester Research Ethics Committee in the UK. All participants, including youth, were aged 16 years and over and provided written informed consent. We utilized thematic analysis, particularly its codebook form, which theoretically allows for conflation of inductive and deductive coding processes, whilst maintaining the value of analyst collaboration and dialogue (15). Data were integrated, initially coded by one researcher and revisited by a second coder. One further independent researcher helped resolve any discrepancies.

## Results

The established themes and subthemes are described below in relation to the research questions on intergenerational factors that can facilitate mental health promotion, with supporting participants’ quotes.

### Theme 1: process of intergenerational learning

Sharing stories of trauma and resilience helped participants to understand and make links with emotions like sadness or anger, which could in turn feed into mental health promotion. The first step to shared learning was the acceptance that each generation could make a valid contribution to individual and collective mental health and wellbeing.

‘I would say age doesn’t matter in front of knowledge, because everyone has different experiences. We should try to learn from everyone.’ Mother 4

‘I don’t think there is any age limit for you to sit with any generation, as long as you are learning something good.’ Youth (female) 9

Such acceptance was more likely to enhance understanding of adversity experienced by different generations, and how they had responded, which could thus inform coping strategies and mental health self-care.

‘The thing that stood out to me was that every generation has its own trauma. Every generation has dealt with their own trauma in their own way.’ Peer Educator 12

‘What I have learnt is that children nowadays are really different from what we were as kids, so I believe they should be brought up and treated as required today.’ Teacher 3

Peer educators appeared to learn from this process by listening to older generation narratives and challenging their own pre-conceptions, and this enhanced their confidence as mental health promotion facilitators. This knowledge iteratively influenced facilitation, as they focused more on intergenerational commonalities rather than differences.

‘Many people believe that our generation is facing more challenges and problems than our parents and grandparents did. However, it’s important to note that our predecessors may not have experienced the same issues that we are currently dealing with. Throughout these discussions, I came to the realization that the problems we face are simply different, not necessarily more or less significant.’ Peer Educator 13

Improved understanding of experiences and needs was linked to emotional literacy, which is a key mechanism in promoting positive mental health. Expression of emotions was related to life circumstances, followed by potential solutions through stronger family relationships and communication.

‘Nowadays the younger generations around us are very aggressive and show a lack of patience. We need such activities for them.’ Mother 10

‘Today, children say that they are stressed and depressed. Before, there was a joint family system. . .there were people to talk to each other. Now we are sitting in one room. But we are all different. Everyone is on their mobile. We are not together, even if we are sitting together.’ Teacher 2

Several youth expressed the wish to improve relational emotional literacy, by sharing knowledge at home. They expressed confidence that they could engage adults in finding common approaches.

‘And I will share it with my mother. How you can express your feelings. And how you can handle them.’ Youth (female) 1

‘It will be difficult to share it with my mother. There is an age difference, but when I share it with them, they respond to it. . .when I talk to my mother, the whole family is present. I tell them that we went to the session. We learned a lot there. Everyone should feel this way. When and where to get angry. How to control.’ Youth (female) 2

During the mental health promotion workshops, intergenerational activities were positively perceived in actively involving participants and putting key messages across. Such activities helped participants view or relate differently to other generations in relation to building resilience.

‘The activity I will remember is when we wrote different messages for the next generation. I enjoyed being an advisor for our next generation. I have an imagination that some years later I will be grown up too and will be seeing another generation growing in front of me.’ Youth (male) 11

‘From today’s activity, I learned that we are a light for our children. We can be a better guide for our children.’ Mother 5

Mental health promotion activities helped convey complex messages in a simple way that could be retained and replicated. They served as icebreakers and involved participants, especially older adults, who might not have had the opportunity to contribute their opinions before.

‘The pot we gave to them represents a heart. And if we’re saying, their heart is full and then they can transfer their knowledge and their wisdom to a younger generation like us, because our heart is empty.’ Peer Educator 11

‘The activities you did with us are really simple such as clay activity, this is everyone’s childhood activity. This activity is best for older people, they will love it. They can re-live their childhood.’ Teacher 17

Participants proposed additional activities for future mental health promotion events or to share with their families. Attention was given on how to also draw male adults, who had not been involved in the workshops.

‘Also, kite-flying because I learned kite-flying from my father. These are some activities which everyone did in their childhood.’ Youth (male) 12

Peer educators’ facilitative role was important in engaging participants and conveying mental health promotion messages. They found it easier to relate to their own age group, whilst some adults projected on the peer educators in relation to their own daughters or granddaughters.

‘I learned a lot from these young girls [peer educators]. Genuinely, the experience was heart-wrenching, as I was considering them as my daughters, the way we teach our daughters and learn from them, and share or even disagree, we did it here. I believe it was helpful.’ Mother 6

‘Especially because people around my age had a smooth relationship, the conversation flowed smoothly. Because they were the same age, they also felt that it was not a big deal. I think it was a relatable factor.’ Peer Educator 6

### Theme 2: knowledge transfer and application

Participants, including peer educators, reported that they had used intergenerational knowledge in several ways following the mental health promotion workshops. Applications involved promoting their wellbeing, developing coping strategies, implementing positive parenting, and challenging gender stereotypes.

‘Before coming here and before today’s awareness session, my thoughts were that I am the caretaker, I am a mother, I am a father, I am a friend, but never thought I was a human too.’ Mother 10

In developing coping strategies, participants drew commonalities across historical periods and related to their life contexts. This was viewed as an opportunity to initiate conversations within their family.

‘Listening to my grandmother would be interesting for others and listening to others will be fun for me.’ Mother 9

‘There will be a generation gap, but the younger generation will adjust with the older generation. And the older generation will adjust with the younger generation.’ Teacher 4

Although mental health promotion workshops were delivered to separate age groups, peer educators co-facilitated more than one workshop, were thus able to contrast generational influences on how they perceived and processed key messages.

‘And they are transferring those dealing strategies to the next generation. They are transferring coping strategies along with trauma. It’s not like the trauma is being transferred.’ Peer Educator 12

‘My observation in both the events, specifically in the girls’ event, was that the coping strategies of the mothers, which had a lot of religious aspects, were the same in the girls’ event. So, my observation was that the mothers transferred their religious coping factors to their children.’ Peer Educator 6

Some participants translated intergenerational perspectives of adversity and how these could be used by different generations in promoting mental health. Others focused more on understanding differences and underpinning reasons.

‘We face the burden of studies and expectations of our parents and schools, which often leads to different issues in the future.’ Youth (male) 11

‘The older generation is very busy. They are not paying attention to their children. I have noticed that the parents have left their children. They are very lacking. . .I have noticed this difference between the younger generation and the older generation, nowadays.’ Teacher 1

To break this cycle, participants endorsed positive parenting and family approaches that involved nurturing, empathy, listening and communication. These attributes should also apply to the extended family.

‘When he [10-year-old son] comes to me with a glass of water, I automatically feel better. If I shout at him too, if I don’t listen to him, he is going to be the same.’ Mother 8

‘If you talk to the younger generation with love, they will be fine with you. If you show anger, they will be rude to you.’ Teacher 3

Considerations of how mental health could be improved often focused on gender issues, especially women’s exclusion from education, employment and social activities. Nevertheless, participants also shared stories of mothers supporting their daughters to access opportunities. This was a positive surprise for peer educators, who were all female and had received professional education. Stories of women’s resilience inspired participants to enhance girls’ opportunities.

‘I appreciated that mothers were given chance to learn and grow. This is rare in our marginalized communities. Never ever the mothers were given a chance to groom, they were considered housewives only.’ Mother 1

‘Even when we spoke to the mothers, they were so encouraging about how they want to focus on education. How they want to teach their daughters and children. That was a bit surprising for me. I didn’t think education would be their priority. . .especially the ladies who have grown up not getting educated. . .and they want to empower the girls.’ Peer Educator 12

‘I will make a habit of small discussion with my daughter to make her realize I am with her. My sister too how mothers should be groomed.’ Mother 3

In addition, there was consideration by male youth on gender expectations of certain behaviours, for example boys lacking emotional literacy. This could be one reason behind fathers’ lack of engagement in family conversations. The workshops offered ideas on how to involve fathers in promoting mental health.

‘From birth, we hear that we are stronger than girls and this becomes part of our personality. We listen to this our whole life. “Boys don’t cry”, but today it was a relief to know our emotions matter too. I love the activities and will surely practise them.’ Youth (male) 8

‘Mothers often participate but the father doesn’t do that, so, yes, if it’s on the weekend, we can do a similar event with fathers. Or some activities that we do in pairs, so this way we can learn about our fathers too, especially about their childhood.’ Youth (male) 9

### Theme 3: knowledge mobilization and impact

In addition to the application of knowledge, several participants had either already shared or proposed to share key mental health promotion lessons with their family, peer group or community. These lessons appeared informed by principles of intergenerational learning and resilience.

‘Don’t think like the person is your friend or enemy, elder or younger. Think about the advice or learning given to you.’ Mother 10

‘I will do that with my son, I will teach him to express himself, I am realizing the after-effects that can be seen if we don’t teach our children, especially our son to express our emotions positively. I don’t want his future family to suffer because of his emotional issues. Even though he is a great boy, we don’t know what the future looks like.’ Mother 8

Participants attributed resilience-building meaning to characters from their workshop activities, which they then related to other family members. This appeared to motivate them to engage them further.

‘I will share with my mother; she is my superwoman.’ Mother 9

‘I will share what I have learned today with my family. I will try to find the specific power of a superhero.’ Boy 1

Particular attention was given on how to involve grandparents in mental health promotion. Youth acknowledged the importance of learning from their experiences, but also of how grandparents could improve their own mental health.

‘We can do this painting activity or decorating dolls activity with grandparents, as they must have done this in childhood, and they miss their childhood, as they are grown up. My grandmother told me they used to do wedding of their dolls, same as real weddings that takes place in reality.’ Youth (female) 9

‘My parents, my nephew, niece, my sister-in-law, my whole family. I would recommend all of them, as they are a really important part of my life. I will arrange this at my home for them, it will be a chance for them to learn. In Islam, it is said we should share our knowledge with others. A great gift from my side to my family, a self-care journey in a colourful world.’ Mother 7

Overall, participants from all groups were positive about contributing to future mental health promotion, but they were tentative about their lack of skills and status in their community. They expressed a preference for co-facilitation with professionals, after receiving further training.

‘I can’t train others, as I think I am not qualified enough. Yes, I can arrange it in my area. I can co-facilitate. What I learned today will be helpful to my children and for myself, but training needs knowledge and I am not at that level.’ Mother 9

‘Right now, we are so young, no one will listen to us. We lack basic knowledge, we can co-facilitate, it would be possible with more training.’ Youth (female) 9

## Discussion

The aim of this study was to explore how intergenerational resilience could inform mental health promotion in disadvantaged Pakistani communities that was driven by youth peer educators. The findings thus make a connection between two previous but separate areas of the literature, that is, how intergenerational resilience can protect mental health (3) and how community volunteers such as peer educators can challenge stigma and promote positive mental health (11). The content and delivery approaches were based on consultation with three generations of local families, and workshops were delivered to youth, parent and teacher groups. Participants reported on their experiences of the process of intergenerational learning, application (individually and within family and community) and recommendations for future scaling-up and enhanced reach. These inter-linked themes indicate the need for the development of a fluid iterative process to move from knowledge acquisition to knowledge transfer, and to knowledge mobilization and impact. Shared narratives were valued by all groups, including peer educators. Participants appeared to identify commonalities in trauma exposure and response across sociocultural and historical contexts, which enabled them to translate to current circumstances. In doing so, they first had to challenge previously held beliefs on age, gender or socioeconomic status. Participants offered examples of how sources of potential intergenerational divides, such as mobile phones fragmenting family ties and reducing communication, could be reframed to strengthen resilience, in this example by using digital technology to bring families together.

This process also presented with multiple challenges that required supported facilitation, especially for community members without professional experience. Mixing generational groups for time-limited workshops, whilst touching upon topics of trauma and mental health, carries certain risks of tensions, challenges of cultural beliefs and even re-traumatization. Facilitation thus requires confidence, competence and skill, and programmes need to be focused, with clear objectives within the remit of promotion rather than intervention to individual mental health needs. Co-production with community stakeholders offers valuable insight to historical trauma and resilience, and traditional perceptions that connect past and present, as well as support systems for signposting. Intergenerational creative activities were favoured by all groups, as these helped to break down barriers, encourage participation, maintain boundaries and fidelity to workshop objectives, and relate complex and sensitive messages.

Mental health promotion requires wider systemic changes of community empowerment and ownership, and direct links with services, so that care pathways are seamless. To this effect, there should be clear links between peer educators and professionals, and demarcation between awareness, prevention, first-line and specialist response. This is particularly important in MWCs, because of misconceptions around mental health and limited capacity. Resource constraints can, however, be compensated by informal psychosocial networks. These are usually viewed as the first source of help, because of trust, in contrast to structural support delivered by professionals. Youth can meaningfully engage and involve their peer group because of existing connections, trust and creativity, as demonstrated by previous studies (16). They can play an active role in promoting mental health awareness in schools and communities, by conveying messages through sports, recreational or social activities. With more active support, training and integration into service systems, they can extend their role to peer support through advocacy, psychoeducation and guidance on developing coping strategies. For these reasons, youth should be central to the design and delivery of mental health promotion programmes for their generation, as well as conduits for other generations, if they are given the right kind of encouragement, tools and space to do so.

Intergenerational resilience narratives should also feed into professional training, as highlighted by previous research on healing by indigenous communities (10). These stages of healing or trauma re-processing have parallels with the three established themes of this study on intergenerational process, transfer and mobilization. Families could be involved as advisers, as in the first phase of this study (12), or as co-facilitators of mental health promotion. This would help peer educators and professionals in engaging communities and enhancing access. Co-facilitation of mental health promotion could strengthen links between communities and services, by complementing intergenerational and mental health knowledge.

These findings and arising implications are compatible with an intergenerational resilience framework that addresses youth mental health across the dynamically linked socioecological systems of youth, families, communities, culture and history (17). Mental health promotion should, therefore, concurrently address different age and community groups, as their needs, beliefs and solutions are interconnected. Such a multidimensional model that conceptualizes resilience as a process or interaction across space and time has implications for mental health promotion, response and prevention (18). It is particularly relevant to resource-constrained settings, where youth often experience multiple needs, which can best be addressed through collaborative care that enhances individual coping strategies and life skills, positive parenting, interpersonal relationships, school and social inclusion (19).

Certain limitations of this study should also be taken into consideration in interpreting the findings. The participating areas were not necessarily representative of other areas of Pakistan such as rural communities or other MWCs. Participants may have held a more positive outlook to mental health in that they volunteered to attend workshops and focus groups. A different sampling framework may have affected engagement or restricted the objective of the workshops to conceptualization and attitudinal issues rather than consideration of coping strategies, thus requiring more time. There was self-selection in the recruitment of peer educators with professional studies and from a socioeconomic background that did not match the disadvantaged communities at focus. No male participants, other than youth, took part, through both natural recruitment and a decision not to mix genders for cultural reasons. Although grandmothers did participate in one workshop, they were not involved in focus group discussions. Quantitative measures of knowledge and attitudes towards mental health, intergenerational relationships and resilience would have provided evidence on impact across different outcomes. These methodological issues can inform future research. Adapted models could involve older adults and parents, and engage males, as both peer educators and community participants. Younger children could have an important role to play too as, in a previous study of five MWCs, including Pakistan, we found that children as young as eight years could interview older adults to relate stories of trauma and resilience to their own experiences, and initiate intergenerational learning (20).

In conclusion, a multidimensional resilience framework that draws upon intergenerational experiences can usefully inform youth mental health promotion, especially in resource-constrained MWC settings. Co-production with holders of intergenerational knowledge, participatory activities, and co-facilitation by peer educators can engage and empower local communities. For such programmes to be sustainable, they need to be integrated within informal and structural support systems.

## Appendix 1. Intergenerational activities used in mental health promotion workshops

**Table:**

Area	Target group	Intergenerational activity
1	Female youth	Writing heartfelt letters to future selves fosters a powerful connection between the participants’ present and future selves. Through this activity they can view themselves in a whole new way, by acknowledging how far they have come in the journey of healing and how far they want to go. Moreover, a planting activity symbolizes hope for a brighter future. The activity also gives an opportunity to participants to tear the internalized sense of helplessness by making something beautiful happen. As they nurture the planted seeds, this act will serve as a final affirmation of their journey, reminding them of their dreams, and the transformative possibilities that lie ahead.
1	Male youth	Participants engage in an interactive superhero worksheet, during which they get an opportunity to reflect and write all their dreams and the strengths/abilities they would need to realize them. By making use of cartoon superheroes, the idea is to transfer an intergenerational strength to youth, for them to hope and dream for a better future.
1	Mothers	The activity is to make a candle in a jar. This is associated with the concept of parents as a light for their children, despite of all the hurdles, and how they can support them along with their own selves.
1	Grandmothers	Participants are provided with heart-shaped clay pots to paint. These pots serve as vessels to hold colourful beads, representing stories of resilience and advice for future generations. Through this activity, intergenerational wisdom and experiences are transferred and preserved, fostering a sense of continuity and connection between generations.
1	Teachers	Participants are given clay blobs, from which they make a simple pinch pot. The activity symbolizes that, as participants are able to sculpt clay, they also possess the capacity to shape their lives. The activity serves as a reminder that we are architects of our own reality, capable of manifesting and transforming our circumstances, while breaking free from the chains of past trauma. Clay represents the self, and the process of shaping it reflects our ongoing journey of self-improvement.
1	Religious teachers	The activity involved participants expressing their anger and stressors on a piece of paper using crayons, then tearing the paper into tiny parts. Afterward, they were given a new clean sheet to think of a symbol of hope or strength, and the torn pieces were used to decorate this fresh artwork. This transformative art process represents a person’s ability to use their inner strength to cope with difficult situations.
2	Youth (female)	Participants are initially provided with doll-shaped pots. They are asked ‘if they were given this pot, would they want to decorate it in a pretty way or keep it plain? would they want to put nice things in it or trash it? would they keep it somewhere it can fall easily and break or in a safe space?’ Pots symbolize us and how, in the same way, we need to take care of ourselves and ensure we fill ourselves with positive energy. Participants then paint their pots and are asked about what they would want to see changed in the next generation, as well as what they would want to change about themselves as adults.
2	Youth (male)	Participants are given colourful papers, which are cut into the shape of a leaf. They are then asked to write about their future on each leaf, and what changes they want to see in future generations. Participants are asked to hang completed leaves on a ‘tree of hope’.
2	Mothers	Clay heart pots are used to symbolize nurturing. Participants are encouraged to fill the heart pots with colourful pebbles, each representing a story of resilience or advice for the younger generation. This activity emphasizes the importance of sharing experiences and filling each other’s hearts with wisdom and positivity. The empty heart pot represents the older generation, which can be filled with mothers’ advice and positivity.
2	Teachers	Clay is used to make pots, by learning to reform, shape and mould one’s personal life with positivity and strength rather than weakness. Clay symbolizes a child’s personality that can continuously be remoulded and re-shaped. This activity emphasizes the importance of teachers’ roles in a child’s life.
